# Regeneration of Humeral Head Using a 3D Bioprinted Anisotropic Scaffold with Dual Modulation of Endochondral Ossification

**DOI:** 10.1002/advs.202205059

**Published:** 2023-02-08

**Authors:** Tao Li, Zhengjiang Ma, Yuxin Zhang, Zezheng Yang, Wentao Li, Dezhi Lu, Yihao Liu, Lei Qiang, Tianchang Wang, Ya Ren, Wenhao Wang, Hongtao He, Xiaojun Zhou, Yuanqing Mao, Junfeng Zhu, Jinwu Wang, Xiaodong Chen, Kerong Dai

**Affiliations:** ^1^ Shanghai Key Laboratory of Orthopaedic Implant Department of Orthopaedic Surgery Shanghai Ninth People's Hospital Affiliated Shanghai Jiao Tong University School of Medicine 639 Zhizaoju Rd Shanghai 200011 China; ^2^ Department of Orthopaedics Xinhua Hospital affiliated to Shanghai Jiaotong University School of Medicine No. 1665 Kongjiang Road Shanghai 200092 P. R. China; ^3^ Department of Oral Surgery Shanghai Ninth People's Hospital Shanghai Jiao Tong University School of Medicine College of Stomatology Shanghai Jiao Tong University National Center for Stomatology National Clinical Research Center for Oral Diseases Shanghai Key Laboratory of Stomatology Shanghai 200011 China; ^4^ Department of Orthopedics The Fifth People's Hospital of Shanghai Fudan University Minhang District Shanghai 200240 P. R. China; ^5^ School of Medicine Shanghai University Jing An District Shanghai 200444 China; ^6^ Southwest JiaoTong University College of Medicine No. 111 North 1st Section of Second Ring Road Chengdu 610036 China; ^7^ The Third Ward of Department of Orthopedics The Second Hospital of Dalian Medical University No. 467, Zhongshan Road, Shahekou District Dalian Liaoning Province 116000 P. R. China; ^8^ College of Biological Science and Medical Engineering State Key Laboratory for Modification of Chemical Fibers and Polymer Materials Donghua University Shanghai 201620 P. R. China

**Keywords:** 3D bioprinting, biomechanical stimuli, dynamic compression, endochondral ossification, humeral joint

## Abstract

Tissue engineering is theoretically thought to be a promising method for the reconstruction of biological joints, and thus, offers a potential treatment alternative for advanced osteoarthritis. However, to date, no significant progress is made in the regeneration of large biological joints. In the current study, a biomimetic scaffold for rabbit humeral head regeneration consisting of heterogeneous porous architecture, various bioinks, and different hard supporting materials in the cartilage and bone regions is designed and fabricated in one step using 3D bioprinting technology. Furthermore, orchestrated dynamic mechanical stimulus combined with different biochemical cues (parathyroid hormone [PTH] and chemical component hydroxyapatite [HA] in the outer and inner region, respectively) are used for dual regulation of endochondral ossification. Specifically, dynamic mechanical stimulus combined with growth factor PTH in the outer region inhibits endochondral ossification and results in cartilage regeneration, whereas dynamic mechanical stimulus combined with HA in the inner region promotes endochondral ossification and results in efficient subchondral bone regeneration. The strategy established in this study with the dual modulation of endochondral ossification for 3D bioprinted anisotropic scaffolds represents a versatile and scalable approach for repairing large joints.

## Graphical abstract

A 3D bioprinted joint scaffold with anisotropic structures and heterogeneous components is successfully fabricated in one step. Meanwhile, orchestrated biomechanical and biochemical stimuli are applied for the dual modulation of endochondral ossification to regenerate heterogeneous hyaline cartilage and subchondral bone simultaneously. This approach may provide an alternative for the design, fabrication, and regeneration of biomimetic joints.

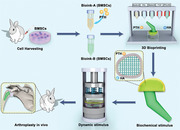

## 1 Introduction

Osteoarthritis is a degenerative joint disease characterized by progressive loss of articular cartilage in synovial joints and affects millions of people worldwide.^[^ ^1^ ^]^ To date, surgical replacement of a diseased joint arthroplasty with a synthetic prosthesis is the only available alternative to restore partial joint function in patients with end‐stage osteoarthritis. Although the surgical procedure is well established, current arthroplasty does not entail biological reconstruction and failures or complications are common.^[^ ^2^ ^]^ Therefore, it is imperative to explore biological methods for the regeneration of biomimetic joints. As an emerging biological technology, tissue engineering may theoretically be used to reconstruct a biomimetic graft with a biological architecture and biofunction similar to those of native joints; thus, tissue engineering is a promising method to replace artificial arthroplasty and treat patients with osteoarthritis.^[^ ^3^ ^]^ A few studies have preliminarily attempted to develop strategies for reconstructing engineered biomimetic joints.^[^ ^4^, ^5^, ^6^, ^7^, ^8^ ^]^ However, these reports were only partially successful and did not achieve satisfactory outcomes in regenerating engineered scaffolds with complex structures comparable to those of native joints. The main difficulties in reconstructing such a biomimetic joint lie in the design and preparation of tissue‐specific scaffolds with the desired biological activity, protocol of induction, and maintenance of hyaline cartilage on the surface, and simultaneous growth rate‐matched formation of large subchondral bone in a single scaffold.

For precise structural fabrication, three dimentional (3D) printing technology has been developed to prepare constructs with ideal geometries and structures to regenerate biomimetic joints.^[^ ^9^, ^10^, ^11^ ^]^ Despite the promise of these constructs, several disadvantages, such as a low bioactive microenvironment, long periods of in vitro cell expansion and seeding, and difficulty in precisely distributing growth factors and seeding cells into scaffolds, may hinder their clinical transition.^[^ ^12^, ^13^ ^]^ Recently, 3D bioprinting technology has received increasing attention in the field of tissue engineering.^[^ ^14^, ^15^, ^16^ ^]^ This technology applies jetting or extrusion approaches to deliver growth factors and living cells into hydrogels, macromolecules, and biomaterials to generate complex 3D biofunctional living tissues or artificial organs.^[^ ^17^ ^]^ In comparison with traditional 3D printing, 3D bioprinting with a high intensity of incorporated cells and better controllability of multitype cells and growth factor distribution can create heterologous bioactive microenvironments in a single scaffold.^[^ ^18^ ^]^ Previous studies have reported that 3D bioprinting can significantly benefit the regeneration of limited osteochondral defects;^[^ ^19^ ^]^ however, the application and feasibility of 3D bioprinting for large joint reconstructions have rarely been explored.

Another tremendous challenge for the regeneration of large joints is the hypertrophy and ossification of the regenerated cartilage tissue, as well as the slow formation of large subchondral bone.^[^ ^20^ ^]^ Bone marrow mesenchymal stem cells (BMSCs) are a promising autologous cell source for bone and cartilage tissue engineering; however, they are associated with a few disadvantages for regenerating large biomimetic joints. First, their ability to regenerate and maintain hyaline cartilage is limited because the default pathway in chondrogenic differentiation of marrow mesenchymal stem cells (MSCs) is terminal differentiation, which results in a hypertrophic phenotype that is a precursor to the process of endochondral ossification.^[^ ^21^, ^22^, ^23^, ^24^, ^25^ ^]^ Second, the strategy of directly inducing MSCs to form a bone‐like matrix within scaffolds, mimicking the embryological process of intramembranous ossification (IMO), often fails to induce large‐scale new bone formation because of poor perfusion and avascular necrosis.^[^ ^26^ ^]^ Endochondral ossification is an indispensable approach for new bone formation; the efficiency of new bone formation is much higher than that of intramembranous osteogenesis and has been advocated as a strategy for large bone formation.^[^ ^20^, ^27^, ^28^, ^29^ ^]^ Thus, the promotion or inhibition of endochondral ossification plays a significant role in the differentiation of BMSCs, and proper modulation of this process may greatly benefit the successful reconstruction of the humeral head.

Spatial regulation by complex signals in the microenvironment, such as molecular factors, matrix structures, and external stimuli, is required for MSCs differentiation.^[^ ^30^ ^]^ Biomechanical stimulation, especially dynamic loading, has recently been developed as a viable strategy for bone and cartilaginous tissue regeneration, mimicking the complex biophysical microenvironment of native osteochondrosis.^[^ ^31^ ^]^ Despite the promise of biomechanical stimulation, dynamic compression of MSCs embedded in scaffolds has been reported to result in highly variable responses from inhibiting the process of endochondral ossification and enhanced chondrogenesis in some studies to promoting endochondral ossification and facilitating osteogenesis.^[^ ^32^, ^33^, ^34^, ^35^ ^]^ To precisely modulate the process in the desired direction, a variety of approaches, such as the proposal of tissue‐engineered constructs that mimic the zonal cartilage organization and extracellular matrix (ECM) composition, the incorporation of various growth factors into specifically prepared hydrogels, or the design of gradient structures with different mechanical properties to regulate the ossification process, have been developed previously.^[^ ^36^ ^]^ However, to date, spatially localized inhibition or promotion of endochondral ossification through different orchestrated symphonies of biomechanical, biochemical, and structural signals resembling the native microenvironment of cartilage and bone for the regeneration of large joints, has not been illustrated.

In the present study, the morphology of a rabbit proximal humeral joint was first captured with laser scanning, and an anatomically correct biomimetic scaffold was designed. Thereafter, specific 3D bioprinting systems with multiple nozzles were used to fabricate rabbit synovial joint scaffolds with different biochemical cues and heterogeneous structures in the outer cartilage and inner bone. Subsequently, a custom‐designed bioreactor was employed for chondrogenic induction and to subject the prepared scaffolds to dynamic compression stimuli. An in vitro analysis was performed to evaluate the combinatorial effect of varied biomechanical compressions and varied structural and biochemical stimuli on the process of endochondral ossification for the reconstruction of anisotropic hyaline cartilage and subchondral bone tissues in large joints. Finally, the total articular surface of the unilateral proximal humeral condyles of skeletal adult rabbits was surgically excised, and arthroplasty was performed using biomimetic scaffolds. Locomotion and weight bearing were analyzed at 4 months to analyze the reestablishment of proper function of the rabbit humeral joint after surgery. Histological analysis of the retrieved humeral head samples, from in vivo experiments, was performed, and the mechanical properties of these samples were assessed. The main content of the current study is shown in the graphical abstract.

## 2 Results

### 2.1 Fabrication of the Artificial Humeral Head

**Figure 1:**
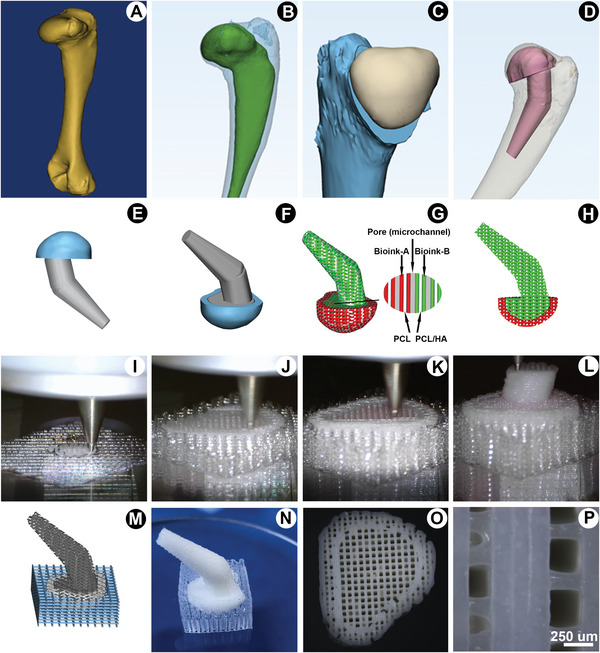
Rabbit humeral head scaffolds with different structures and compositions were constructed based on CAD/CAM technology. A–D) Scan of the left humeral head and effect map of the prosthesis design in New Zealand white rabbits. E–H) Layering the design of the humeral head and printing the path design. I–L) Preparation process for 3D printing of the humeral head. M,N) Print model and a physical object. O,P) Base view and magnified image of the humeral head.

The bioscaffolds were designed according to the 3D data obtained from the computer tomographic (CT) scanning of the rabbit proximal humeral condyle (**Figure** **1**A–C). The designed bioscaffold consisted of an upper mushroom‐like head and lower intramedullary stem for surgical fixation (Figure 1D–F). Figure 1G,H shows the printed model of the designed prosthesis. Next, the organ printing united system (OPUS, Novaprint, Suzhou, China) was applied to fabricate a 3D hemispherical scaffold with dimensions of 12.42 × 10.11 × 16.88 mm^3^ (length × width × height) (Video S1, Supporting information). The printing process is shown in Figure 1I–L. Figure 1M,N shows that the fabricated humeral head shared a precise shape and size with the designed rabbit proximal femoral condyle, while the lower stem showed a perfect match with the marrow cavity. To promote nutrient transportation toward the interior of the scaffold and facilitate the growth of various tissues in different areas, two interconnected microchannels with large pore sizes (250–300 µm) in the inner layer and small pore sizes (100–150 µm) in the outer bone region were designed with a porosity of 54.6 ± 1.2% (Figure 1O). The thickness of the outer layer reached ≈0.6 mm (Figure 1O,P).

### 2.2 Biocompatibility of the Artificial Humeral Head

**Figure 2:**
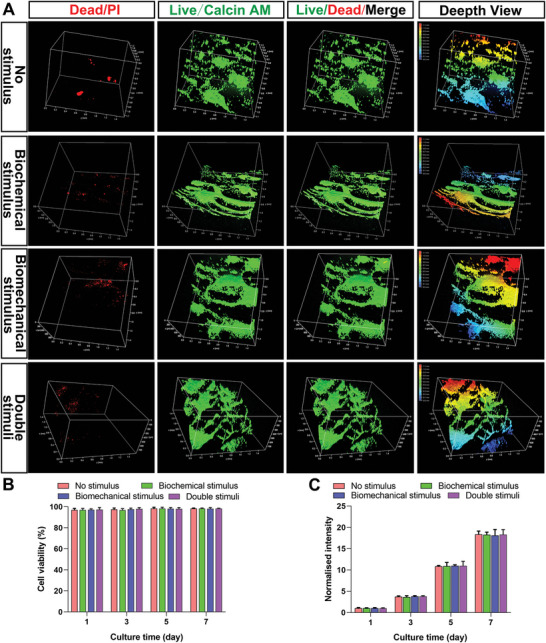
Laser confocal scanning was used to detect the biocompatibility of each stent. A,B) Live/dead staining showed the survival state of cells and the survival rate of cells at 1, 3, 5, and 7 days of stimulation, while C) the CCK‐8 assay showed the proliferation of cells in the scaffold at 1, 3, 5, and 7 days of stimulation.

Each group of bioprinted scaffolds was stained with live dead cells after 1, 3, 5, and 7 days of cultivation. BMSCs were evenly distributed among the scaffolds (**Figure** **2**A, day 3). Notably, high cell viability with few dead cells (more than 95%) was observed in the different scaffolds, and statistical analysis demonstrated no significant difference among the groups (Figure 2B). In addition, the cell count kit‐8 (CCK‐8) assay showed that cell proliferation increased with culture time. Moreover, the normalized optical density (OD) values were not significantly different among the four groups at the selected time points (1, 3, 5, and 7 days) (Figure 2C). These results indicated the excellent biocompatibility of the fabricated humeral head scaffolds for long‐term cell growth.

### 2.3 Endochondral Ossification Related Genes Expression after In Vitro Stimulation

**Figure 3:**
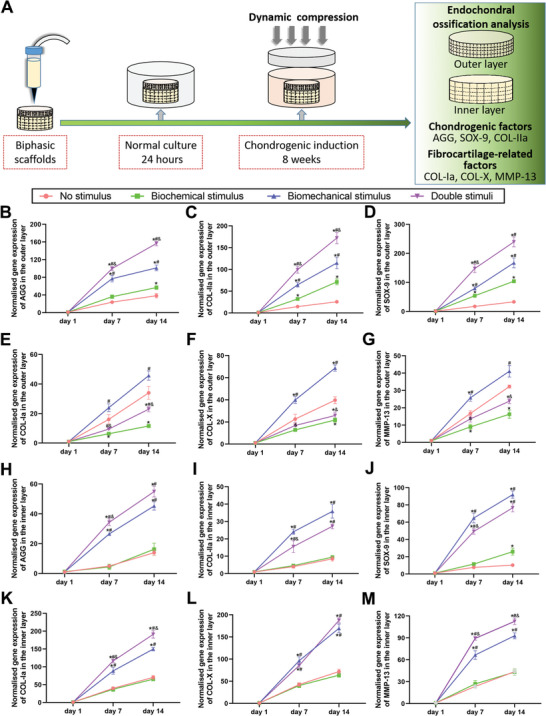
Gene expression related to the process of endochondral ossification in the upper cartilage layer and the lower subchondral bone layer of the simulated cylindrical scaffolds. A) Schematic diagram of the stimulation process. The expression levels of the hyaline cartilagerelated marker genes AGG, COL‐IIa, SOX‐9 and fibrocartilage‐related genes COL‐1a, COL‐X, and MMP‐13 in the outer cartilage (B–G) and inner bone areas (H–M) in each stimulation group. An * indicates a *P*‐value < 0.05 compared with the native humeral group. A # indicates a *P*‐value < 0.05 compared with the biochemical stimulus group. An & indicates a *P*‐value < 0.05 compared with the biomechanical stimulus group.

To evaluate the effect of synergistic dynamic compression stimulation with parathyroid hormone (PTH) loading on chondrogenesis and endochondral ossification of BMSCs in different scaffolds, reverse transcription‐polymerase chain reaction (RT‐PCR) was carried out to evaluate the expression of hyaline cartilage‐related genes (collagen‐II (COL‐II), aggrecan (AGG), and Sry related HMG box‐9 (SOX9)) and fibrocartilage‐related genes (collagen‐I (COL‐I), collagen‐X (COL‐X), and matrix metalloproteinase‐13 (MMP‐13)) in different scaffolds after incubation for 1, 7, and 14 days in vitro (**Figure** **3**A). As shown in Figure 3, in the upper layer, the expression of hyaline cartilage‐related genes was the highest (Figure 3B–D) in the double stimuli scaffolds when compared with that of the other three groups. Notably, the promotion of hyaline cartilage‐related genes was significantly better under biomechanical stimulus than under biochemical stimulus; both were more prominent than that under no stimulus. Meanwhile, the expression of fibrotic cartilage genes (COL‐I, COL‐X, and MMP‐13) in the upper layer was most pronounced in the biomechanical stimulus groups, followed by the no stimulus and double stimuli groups, and was last in the biochemical stimulus group (Figure 3E–G). The above results showed that although dynamic compression promoted the chondrogenic differentiation of BMSCs, it stimulated the endochondral ossification process simultaneously, while the combination of PTH and dynamic compression not only promoted the chondrogenesis of BMSCs but also inhibited endochondral ossification. In the lower layer, the expression of hyaline cartilage‐related genes (Figure 3H–J) and fibrocartilage‐related genes (Figure 3K–M) in the biomechanical stimulus and double stimuli groups were much higher than those in the no stimulus and biochemical groups, with the highest expression of fibrocartilage genes (COL‐I, COL‐X, and MMP‐13) in the double stimuli group at 14 days. Furthermore, we analyzed the expression of osteogenesis‐related genes, such as osteopontin (OPN), runt‐related transcription factor‐2 (RUNX‐2), and osteocalcin (OCN) (Figure S1, Supporting Information), in BMSCs in the lower layer from different scaffolds. Similarly, compared with that in the no stimulus and biochemical stimulus groups, the expression of osteogenesis‐related genes was higher in the biomechanical group. Synergistic dynamic compression stimulation with PTH loading in double stimuli scaffolds further promoted the expression of the above‐mentioned genes (Figure S1, Supporting Information). These results indicate that the double stimuli scaffolds of dynamic mechanical compression combined with PTH could better induce chondrogenic differentiation of BMSCs and inhibit endochondral ossification, while the combination of dynamic mechanical compression and hydroxylapatite (HA) stimulated the developmental process of endochondral ossification and benefited the osteogenesis of BMSCs in vitro.

### 2.4 Immunofluorescence of Endochondral Ossification‐Related Markers after In Vitro Stimulation

**Figure 4:**
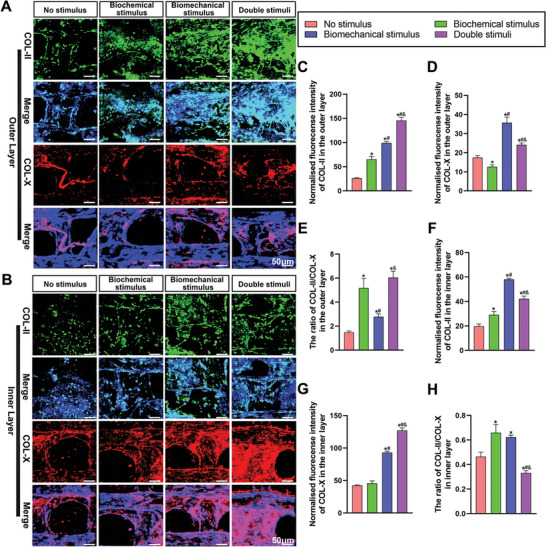
Immunofluorescence analysis of COL‐II and COL‐X after in vitro stimulation. Immunofluorescence images of COL‐II and COL‐X in the outer layer (A) and the inner layer (B) of the no stimulus group, biochemical stimulus group, biomechanical stimulus group, and double stimuli group. Quantitative analysis of COL‐II, COL‐X, and COL‐II/COL‐X expression in the outer layer (C–E) and the inner layer (F–H) of the four different groups. An * indicates a *P*‐value < 0.05 compared with the native humeral group. A # indicates a *P*‐value < 0.05 compared with the biochemical stimulus group. An & indicates a *P*‐value < 0.05 compared with the biomechanical stimulus group.

To further evaluate the effects of biological 3D printed humeral head scaffolds in modulating the process of endochondral ossification of BMSCs at the protein level, we performed immunofluorescence staining for COL‐II and COL‐X in both the outer and inner layers of different scaffolds after culturing in vitro for 30 days. As shown in **Figure** **4**, for the outer cartilage area, compared to the no stimulus group, the immunofluorescence signifying COL‐II was most prominent in the double stimuli group, followed by the biomechanical and biochemical stimulus groups (Figure 4A). COL‐X showed an almost reversed trend. Interestingly, the expression of COL‐X in the biomechanical stimulus group was much stronger than that in the biochemical stimulus and double stimuli groups (Figure 4A). The quantification of immunofluorescence intensity confirmed this observation with the ratio of COL‐II/COL‐X being highest in the double stimuli group and lowest in the no stimulus group (Figure 4C–E). In the inner layer, the relative expression of COL‐II in each group was statistically lower than that in the upper layer, whereas the expression of COL‐X was much more pronounced (Figure 4B). Specifically, the expression of COL‐II was the highest in the biomechanical stimulus group, followed by the double stimuli and the biochemical stimulus groups, which were both much more intensive than that of the no stimulus group (Figure 4B,F). COL‐X expression was highest in the double stimuli group in comparison with the other three groups. The intensity of COL‐X in the biomechanical stimulus group was only second to the double stimuli group and was much higher than in groups without dynamic compression (no stimulus group and biochemical group) (Figure 4B,G). The ratio of COL‐II/COL‐X in the inner layer was lowest in the double stimuli group, followed by no stimulus, the biomechanical and biochemical stimulus groups (Figure 4H). The above results are consistent with the gene analysis and indicate superior inhibition of endochondral ossification and chondrogenic ability of BMSCs under the synergistic stimulation of dynamic compression with PTH, and the significant promotion of the development of endochondral ossification under the combination of biomechanical stimulation and HA induction.

### 2.5 Histological Evaluation of Chondrogenic Differentiation of BMSCs after In Vitro Stimulation

After in vitro stimulation for two months, gross and microscopic views of different scaffolds were observed to further test the treatment effect of different stimuli on cartilage and bone regeneration. As Figure S2, Supporting Information, demonstrates, the outer layer of the no‐stimulus group was rough, and no obvious tissues were formed. In contrast, various degrees of pink, half‐transparent, and new tissue were observed in the other three groups. Among the stimulated scaffolds, a continuous and tough layer of tissue covered the humeral head surface in the double stimuli group. In addition, the tissue thickness in the double stimuli group was much thicker than that in the other two groups.

**Figure 5:**
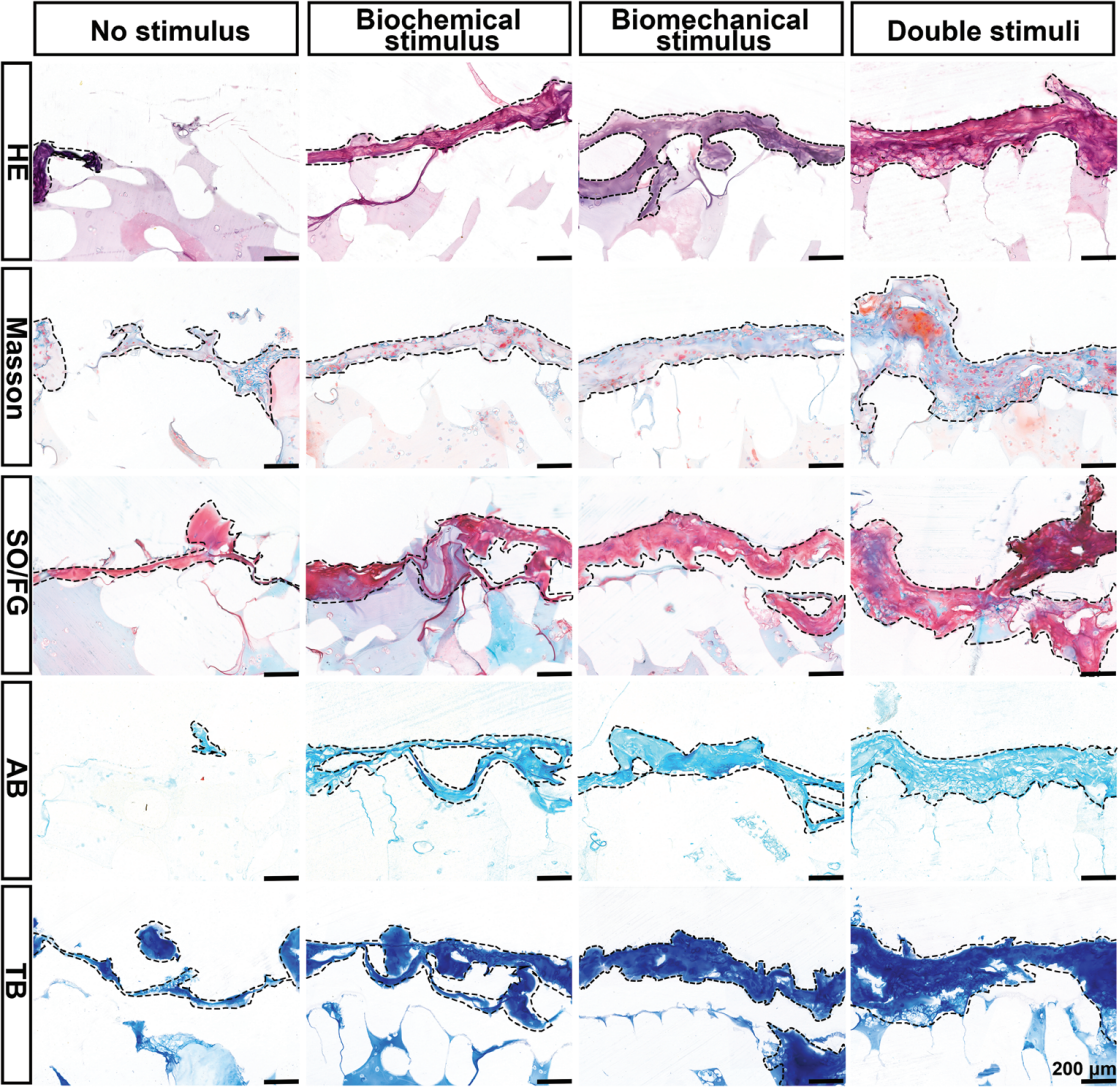
Histological examination of the hard tissue sections of the humeral head after in vitro stimulation in each group. HE staining, Masson, SO/FG, AB staining, and TB staining.

Continuous and homogeneous hyaline cartilage regeneration significantly contributes to the function of the regenerated biological joints. To further evaluate the facilitatory effect of different stimuli on cartilage regeneration, histological evaluations, such as hematoxylin‐eosin staining (HE),  Masson–Trichrome staining (Masson), safranin O/fast green staining (SO/FG), alcian blue staining (AB), and toluidine blue staining (TB), were performed. As shown by the histological analysis (**Figure** **5**), only a small amount of scattered cartilage tissue was visible on the surface of the non‐stimulated group. In contrast, the staining demonstrated that the thickness and coverage of cartilage tissue generated on the surface of the scaffolds gradually increased in the biochemical, biomechanical, and double stimuli groups. The surface layer of the double stimuli group regenerated thick, continuous cartilage with better ECM deposition, which was evidenced by more cartilaginous matrix production as revealed by SO/FG staining and the better cell filling demonstrated by HE and Masson staining. In comparison, thinner cartilage was observed in the biochemical and biomechanical stimulus groups. In the no‐stimulus group, little was isolated or intermittent. Statistical analysis confirmed the above results (Figure S3, Supporting Information). It is worth noting that despite the outer layer of scaffolds being primarily designed to be 0.6 mm, the histological evaluation showed that the cartilage tissue produced by the different groups after in vitro stimulation just ranged from 15 (no stimulus group) to 240 µm (double stimuli group). Meanwhile, most of the cartilage tissue was generated on the scaffolds′ surface with little observed in the outer layer. This may be due to tissue collapse during the histological process and the occupation of the PCL pillars. The above results indicate that dynamic mechanical compression combined with PTH stimulation could synergistically promote the formation of cartilage tissue on the surface of humeral head scaffolds.

### 2.6 Radiology and Gait Evaluation of 3D Bioprinted Humeral Head Restoration In Vivo

**Figure 6:**
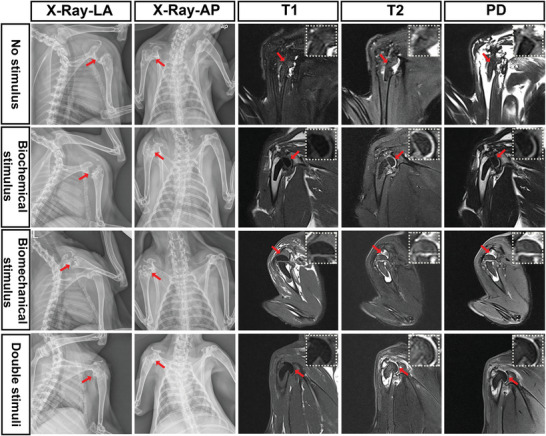
X‐ray and MRI photos of the shoulder joint at 4 months after humeral head arthroplasty with fabricated scaffolds in each group. LA: lateral view, AP: anterior and posterior view, T1: T1 weighted imaging, T2: T1 weighted imaging, PD: proton density‐weighted image.

X‐ray and MRI scans of the shoulder joints were performed to evaluate the fixation of the implanted humeral head restoration and formation of regenerated tissue (**Figure** **6**). Four months after the implantation of the humeral head prosthesis, anterior‐posterior (AP) and lateral (LA) X‐rays showed that a high density of regenerated new bone at the top of the excised humeral stem was most prominent in the double stimuli group, followed by the biomechanical stimulus group and biochemical stimulus group, and was least prominent in the no stimulus group. Additionally, X‐ray images demonstrated that the regenerated humeral head in the double stimuli group showed a regular shape similar to that of the contralateral native head. The results showed that there was a significantly larger volume of newly formed bone in the double stimuli group than in the other three groups (Figure 6). In addition, MRI confirmed that all implanted prostheses remained in the joint with no dislocation. However, the prosthesis was broken in the no‐stimulus group, and only the stem remained in the medullary cavity of the humerus. In comparison, the prostheses in the other three groups were intact and remained in position. Furthermore, as shown by MRI, the newly regenerated cartilage tissue was thicker in the double stimuli group than in the biomechanical and biochemical groups. In addition, we compared the normal humeral head and blank control that underwent arthroplasty without scaffold implantation. As shown in Figure S4, Supporting Information, demonstrates, there were large areas of inflammatory edema in the medullary cavity in the blank control group, without evident new bone or cartilage tissue regeneration. The locomotion of the operated rabbits was also evaluated, and the results showed that 1 month after surgery, the movements of rabbits from the double stimuli group involved only a little grip (Video S2, Supporting information). At 4 months post‐operation, the movements of rabbits in the double stimuli group significantly improved and were similar to those of normal rabbits without detectable deformation (Video S3, Supporting information).

### 2.7 Inflammatory, Histological, and Mechanical Evaluation of the Regenerated Humeral Head In Vivo

Quantification of inflammatory factors in the serum (interleukin‐1 beta [IL‐1*β*] and tumor necrosis factor‐alpha [TNF‐*α*]) of surgical rabbits was performed (Figure S5, Supporting Information). In the first week after implantation, the two factors in all four groups remained at high levels, which is considered a normal acute inflammatory response;^[^ ^37^ ^]^ however, the levels of both cytokines gradually decreased and remained at a relatively low level from 2–12 weeks after implantation. Although a comparison with the healthy normal group was not performed, the decrease in the expression of the two main inflammatory cytokines indirectly suggested that the inflammatory reaction alleviated with time. Notably, the decrease in inflammatory factors in the double stimuli group was more prominent than that in the other groups. TNF‐*α* levels in the three stimulus groups were significantly lower than those in the no‐stimulus group 2 weeks after implantation. Furthermore, the level of TNF‐*α* in response to the double stimuli was the lowest 4 weeks after implantation. The trend of IL‐1*β* expression was similar to that of TNF‐*α*, and the IL‐1*β* level was statistically lower in the double stimuli group than in the other three groups as early as two weeks after in vivo implantation.

**Figure 7:**
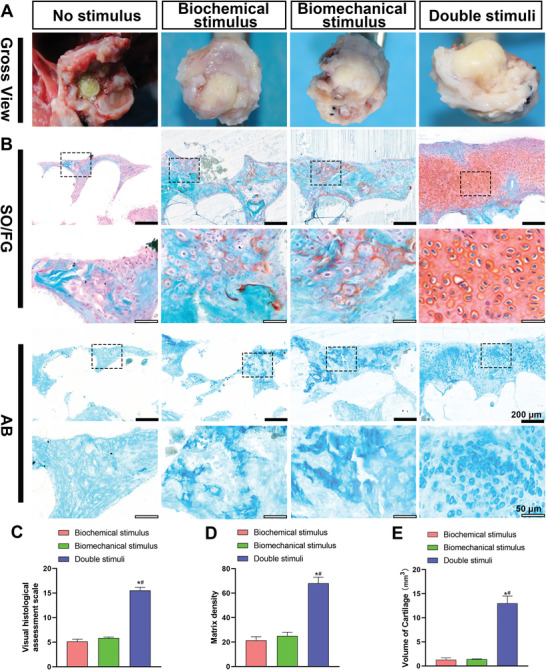
Histological examination of the cartilage regeneration in the humeral head after in vivo semi‐shoulder arthroplasty. A) The gross view of humeral head specimens in vivo after 4 months of shoulder arthroplasty. B) The detection of chondrogenesis with SO/FG staining and AB staining. The visual histological assessment scale (C), the matrix density (D), and the volume of cartilage (E) in the biochemical stimulus group, biomechanical stimulus group, and double stimuli group. The * indicates a *p*‐value < 0.05 compared with the biochemical stimulus group. A # indicates a *p*‐value < 0.05 compared with the biomechanical stimulus group. The no stimulus group is excluded from statistical analysis.

**Figure 8:**
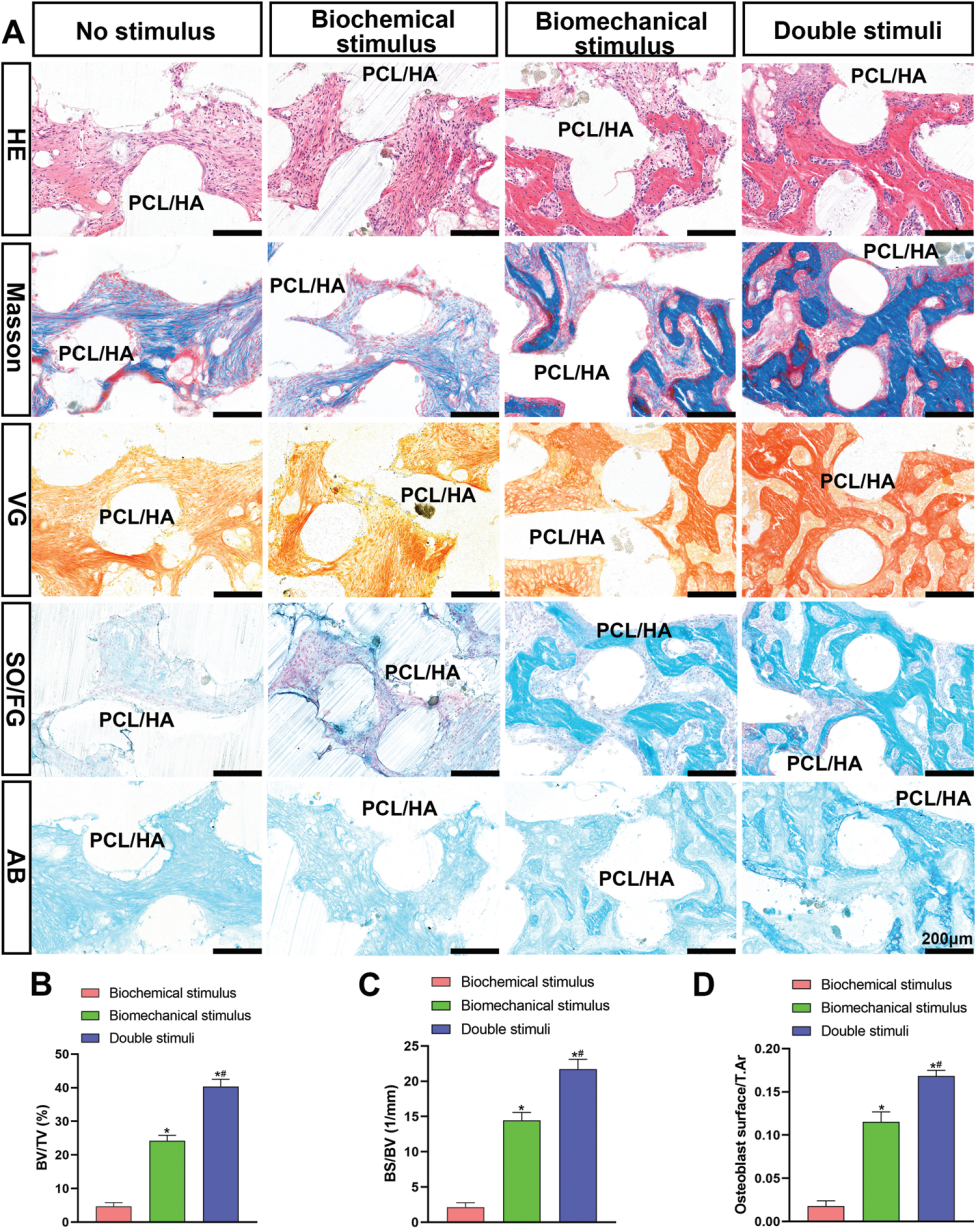
Histological examination of the subchondral bone regeneration in the humeral head after in vivo semi‐shoulder arthroplasty. A) HE, Masson, and VG staining of the subchondral bone area in each group after in vivo implantation. B) The BV/TV, C) BS/BV, and D) the osteoblast surface/T.Ar. An * indicates a *p*‐value < 0.05 compared with the biochemical stimulus group. A # indicates a *p*‐value < 0.05 compared with the biomechanical stimulus group. The no stimulus group is excluded from statistical analysis.

To further verify cartilage and bone formation in the regenerated humeral head, the experimental animals were euthanized at 4 months after arthroplasty, and specimens were collected for gross viewing. As shown in **Figure** **7**A, in the no‐stimulus group, the head and stem of the humeral head scaffold were detached and a residual humeral stem scaffold was observed in the medullary cavity (the residual fragmentary humeral head scaffold was used for histological staining). In the biochemical and biomechanical stimulus groups, a small area of white translucent cartilage‐like tissue was visible on the humeral head scaffold surface, while in the double stimuli group, the shape of the humeral head scaffold was complete and similar to that of the native humeral head, with a large amount of smooth and white translucent cartilage‐like tissue on the surface. SO/FG and AB staining (Figure 7B) showed that the tissue regeneration area on the surface of the double stimuli group was higher than that of the other groups. Additionally, most of the area of the outer layer was SO‐positive, and only a few areas were stained with FG. Notably, the regenerated cartilage tissue surface in the double stimuli group was more uniform, smooth, and continuous than that in the other two groups. For histological analysis, the visual histological assessment scale (Figure 7C), the matrix density (Figure 7D), and the volume of cartilage (Figure 7E) were highest in the double stimuli group when compared with the other two stimulus groups. In the biochemical and biomechanical stimulus groups, the volume of newly regenerated cartilage tissue on the surface area of the scaffold, visual histological assessment scale, and matrix density showed no significant difference and were secondary to the double stimuli group (Figure 7C–E). In addition, only a few SO‐positive areas were observed. In the no‐stimulus group, the results showed that most of the newly formed tissue in the residual humeral head scaffolds was fibrotic tissue, with little cartilage and bone regeneration. In the subchondral area, HE, Masson, Van Gieson (VG), SO/FG, AB and TB staining revealed that the amount of regenerated bone was highest in the double stimuli group, followed by the biomechanical group, biochemical group (**Figure** **8**A), and no stimulus group. Quantitative analysis of bone volume/total volume ratio (BV/TV), bone surface/bone volume ratio (BS/BV), and the number of osteoblasts, further verified this trend (Figure 8B–D). Immunofluorescence staining (Figure S6, Supporting Information) demonstrated that the double stimuli group had the most COL‐II‐positive areas, while COL‐X‐positive areas were the least abundant. This finding indicated that the two stimuli significantly promoted chondrogenic differentiation of BMSCs and effectively prevented chondrocyte hypertrophy. These results showed that biomechanical stimulation promoted the endochondral ossification process of regenerated cartilage tissue and accelerated the formation of mature bone tissue.

Compression destruction of the regenerated humeral head was performed to test the repair effect of different scaffolds. For the joints retracted from the double stimuli group, the maximum mechanical failure load was close to that of a normal humeral head and statistically higher than those of the joints processed in the biochemical and biomechanical groups. In addition, the maximum load value in the biomechanical group was significantly higher than that in the biochemical group (Figure S7, Supporting Information). These results further confirmed the results of histological staining. These results suggest that, compared to the scaffolds from the other three groups, the double stimuli scaffold not only demonstrated better cartilage and bone regeneration capacity but also better preserved joint function with a lower intra‐articular inflammatory reaction after in vivo implantation.

## 3 Discussion

Tissue engineering of biological joints shows potential for the treatment of advanced osteoarthritis.^[^ ^3^ ^]^ Nevertheless, to date, no significant achievements have been achieved in reconstructing a large tissue‐engineered joint with a functional hyaline cartilage layer and well‐matched subchondral bone.^[^ ^4^, ^5^, ^6^, ^7^, ^8^ ^]^ In the present study, we used a 3D bioprinting technique to fabricate a biomimetic humeral head with anisotropic structures and heterogeneous components through OPUS in a single step. The fabricated scaffolds mimicked the native biphasic structure and composition of joint cartilage and subchondral bone. Furthermore, we studied the effect of dynamic mechanical cues combined with biochemical cues ( PTH or chemical component HA) on endochondral ossification progress and regeneration of large joint cartilage and bone. The results showed that the combination of dynamic mechanical stimulation and PTH inhibited the endochondral ossification process and promoted hyaline cartilage regeneration. In contrast, the combination of dynamic compression and the chemical component HA accelerates the process of endochondral ossification, which increases the efficiency of large‐scale bone regeneration. After arthroplasty of the shoulder joint with the prepared scaffolds, the upper limbs of the rabbits regained complete function. Imaging and histological examination results confirmed that a biological neohumeral head with ideal mechanical properties was rebuilt during the long‐term follow‐up. Our study provides insights into the successful reconstruction of biological joints for future clinical applications.

Cartilage plays an important role in the biofunction of large joints, and regeneration of articular cartilage through tissue engineering remains a tremendous challenge in bone tissue engineering.^[^ ^38^ ^]^ The interaction of MSCs with their environmental cues has been reported to play a significant role in MSC differentiation,^[^ ^39^, ^40^ ^]^ and dynamic compression appears to be one of the most important stimuli for MSC chondrogenic differentiation. Dynamic loading alone has been found to delay the process of endochondral ossification of chondrogenic MSCs, but it is not sufficient to inhibit hypertrophy. Rapid upregulation of hypertrophy‐related markers has been frequently observed during chondrogenic differentiation of BMSCs.^[^ ^41^, ^42^, ^43^ ^]^ Interestingly, in these reports, with a combination of growth factors, such as TGF‐*β*3 and dynamic compression, a stable chondrogenic phenotype with limited induction of fibrocartilage and hypertrophic markers (COL‐I and COL‐X) was obtained. These findings were consistent with our results. In this study, dynamic compression stimulation was applied to promote cartilage regeneration of the humeral head bionic joint scaffold. In vitro experiments suggested that pure dynamic compression could statistically promote the expression of COL‐II, AGG, and SOX‐9 compared with the no‐stimulus group; however, pure dynamic compression could not maintain a stable hyaline phenotype, with significantly upregulated expression of fibrotic cartilage‐related genes, such as COL‐X, COL‐I, and MMP‐13, as the cultivation progressed. The in vivo results for the pure dynamic stimuli group further verified that dynamic stimuli alone were insufficient to prevent endochondral ossification in the outer layer of the fabricated scaffolds, with most of the newly regenerated cartilage ossified. To maintain the regeneration of hyaline cartilage, we incorporated it into the cartilage bioink as a biological cue. Studies have shown that PTH inhibits hypertrophy and calcification of chondrocytes to prevent endochondral ossification of hyaline cartilage.^[^ ^44^, ^45^, ^46^, ^47^, ^48^ ^]^ Zhang et al. injected exogenous PTH into the joint cavity in situ and found that the quality of cartilage repair was significantly improved.^[^ ^49^ ^]^ Zheng et al. reported that mechanical stress stimulates the transport of parathyroid hormone 1 receptor (PTH1R) to the cilia and activates PTH signaling in nucleus pulposus (NP) cells, which enhances the transcription of the TGF‐*β*‐connective tissue growth factor (CCN2)‐matrix protein signaling cascade and helps maintain aging intervertebral disc homeostasis. Meanwhile, intermittent injection of PTH could significantly attenuate disc degeneration in aged mice and improve intervertebral disc height and volume by increasing the levels of TGF‐*β* activity, CCN2, and aggrecan.^[^ ^50^ ^]^ In our study, we found that PTH did benefit the preservation of hyaline cartilage formation after induction with MSC chondrogenic medium. The expression of hyaline cartilage‐related markers in the double stimuli group was significantly higher than that in the corresponding groups, while the expression of COL‐1, COL‐X, and MMP‐13, was significantly inhibited and statistically lower than those in the pure dynamical stimulus group and biochemical stimulus group. Furthermore, after the prepared scaffolds were implanted in vivo for 4 months, the results showed that with the synergistic stimulation of biofactors and dynamic compression, the regenerated cartilage preserved the hyaline cartilage appearance and showed a histological appearance similar to that of the native humeral head.

The tissues in living bodies are composed of gradient structures and compositions such as cellular phenotype, ECM composition and structure, and biochemical cytokines.^[^ ^51^, ^52^ ^]^ Compared with other tissues, the gradients are much more evident in load‐bearing osteochondral joints, which possess depth‐wise transitional structures composed of cartilage, calcified cartilage, and subchondral bone in the joints.^[^ ^53^ ^]^ The gradients in ECM composition and the associated cell phenotype in large joints have been attributed to the naturally varying elasticity on the vertical axis.^[^ ^54^ ^]^ Such a gradient in mechanical features played a vital role in minimizing bone–bone impact loading while maintaining the structural integrity of the joint. In the present study, to mimic the native structure of the humeral head, especially the biphasic structure of cartilage and subchondral bone, different materials were applied to construct the framework of 3D bioprinted scaffolds: low‐molecular‐weight pure poly(*ε*‐caprolactone) (PCL) with a lower mechanical modulus in the outer zone and a much stiffer PCL/HA material in the inner zone. In addition, the pore structure of the fabricated humeral head scaffold was designed with two different sizes: a small pore size in the outer cartilage layer and a large pore size in the inner bone layer. These different structures and components contribute to the gradient mechanical distribution in the scaffolds. Under dynamic compression, the deformation of the outer area was expected to be more prominent than that of the inner area. A previous study demonstrated that multiphenotypic differentiation of MSCs occurred in response to different magnitudes of local dynamic compressive strain in the same biochemical environment.^[^ ^55^ ^]^ Specifically, areas of high compressive strain inhibited osteogenic differentiation of MSCs while stimulating chondrogenic differentiation and promoting ECM deposition, resulting in the development of softer cartilaginous tissue.^[^ ^55^ ^]^ In contrast, MSCs in areas with lower magnitudes of compressive strain preferentially differentiated toward an osteogenic lineage, even overriding biochemical cues to provide the groundwork for osteochondral interface tissue.^[^ ^55^, ^56^ ^]^

In addition to the mechanical features, the pure structures of scaffolds have also been deemed to play a significant role in the differentiation of MSCs, and a discrete gradient in the distribution of pore sizes has been suggested to influence MSC osteogenic and chondrogenic differentiation.^[^ ^43^, ^57^, ^58^ ^]^ Andrea et al. reported a gradual increase in chondrogenic markers within gradient structures with decreasing pore size from 580 to 230 µm. This is accompanied by the development of an increasingly compact ECM.^[^ ^59^ ^]^ We previously described the regeneration of anisotropic cartilage by 3D bioprinting with the release of dual‐factor and gradient‐structured constructs.^[^ ^60^ ^]^ The gradually varying pore sizes provided biomechanical cues for the induction of cartilage with varied constitutions that mimicked the native composition of cartilage from fibrocartilage. The gradient structures in our 3D‐bioprinted constructs also contributed to the different differentiation rates of BMSCs. The in vitro results showed that during the early induction period, the expression of biomarkers for hyaline cartilage regeneration (COL‐IIa, GAG, and SOX9) was observed in all scaffolds. However, these factors were more prominent in the outer zone, with a smaller pore size, than in the inner zone, with a large pore size in each group. With the combination of other stimulations, this trend was more obvious. This may result from a related higher cell density in the outer small pore area and the consequent generation of a hypoxic microenvironment in the small pore zone. These outcomes are known to facilitate chondrogenesis.^[^ ^61^, ^62^ ^]^

The highly efficient regeneration of large bones that matches the speed of joint cartilage is another critical issue in reconstructing a biological joint. Traditionally, tissue engineering has focused on directly generating bone by inducing the osteogenic differentiation of MSCs or osteoprogenitor cells, which recapitulates the developmental process of IMO. However, this approach is slow and inefficient, cannot match the speed of newly formed cartilage, and may result in tissue collapse in mechanical loading joints.^[^ ^27^ ^]^ Recently, strategies that recapitulate the developmental process of endochondral ossification by which intermediate cartilage tissues are substituted by bone have received increasing attention in the field of bone tissue engineering.^[^ ^26^, ^63^, ^64^ ^]^ Several studies have confirmed that it is possible to effectively repair large bone defects in animal models using endochondral approaches by which cartilage intermediaries engineered using MSCs act as templates for endochondral bone formation in vivo.^[^ ^26^, ^65^, ^66^, ^67^, ^68^ ^]^ In our study, the subchondral region of the bioprinted scaffolds was immersed in a chondrogenic medium in vitro combined with the stimulation of dynamic compression, which led to the formation of intermediate cartilage tissue. Later, regenerated cartilage templates underwent endochondral ossification under the induction of HA, which has been reported to promote chondrocyte hypertrophy, induce osteocytes, and integrate cartilage repair and subchondral bone.^[^ ^69^ ^]^ Vascularization within hypertrophic cartilage templates is considered a significant process in endochondral ossification, and scaffold architecture may play a vital role in the success of tissue‐engineered endochondral constructs by allowing vessel invasion.^[^ ^26^, ^60^, ^70^ ^]^ In our study, the pores designed in the inner zone of the fabricated joint ranged from 250 to 300 µm, which is beneficial for the formation and invasion of vessels. In addition, the incorporation of microchannels into the inner zone of fabricated scaffolds, according to a previously published method, has been shown to facilitate the diffusion of nutrients and oxygen to incorporated cells, overcome the maximum diffusion limit of 0.1–0.2 mm for cell survival in engineered scaffolds, and benefit the formation and invasion of microvessels.^[^ ^12^ ^]^ Furthermore, hypertrophic chondrocytes are capable of secreting pro‐angiogenic and pro‐osteogenic factors, which in turn bolster the endochondral ossification process and play important roles in both vascularization of constructs and mineralization of the ECM.^[^ ^28^, ^29^ ^]^ The in vivo results further demonstrated that the designed architecture of the present constructs and the biochemical strategy applied in this study improved the efficacy and reproducibility of bone formation, matching the fast speed of cartilage regeneration in large joints.

The strategies applied in the current study would likely encounter some limitations upon implementation before they can be adopted in the clinic. First, we did not identify the specific mechanotransduction pathways involved in the spatial regulation of double stimuli in the developmental process of endochondral ossification of stem cells, which may offer new clues about engineering cartilage and bone tissue with anisotropic structures that resemble those in the native tissue.^[^ ^71^ ^]^ The influence of multiple stimuli on the process of endochondral ossification is complicated, and the identification of single stimuli that modulate this process is challenging. Further studies should use spatial transcriptomics and single‐cell sequencing to achieve this goal. Second, to fabricate the joint biomimetic scaffolds, we classified the humeral head into inner and outer regions as a measure of heterogeneity, but more complex models may be needed to mimic the gradual spatial transition of anisotropic cell phenotypes and ECM compositions of the native joint. Moreover, in the present study, in the in vitro analysis of endochondral ossification modulation by external stimulation, two separate cylindrical scaffolds were used to evaluate related genes and proteins in the outer and inner regions of the humeral scaffolds. Although the scaffolds were stacked together to imitate biomimetic humeral head scaffolds, the morphology was quite different from the curved surface of the native humeral head, and the mechanical loading may differ from that of the compressed fabricated biomimetic scaffolds. Further studies on anatomical biological cartilage and bone scaffolds should be conducted.

## 4 Conclusion

In summary, we demonstrated a 3D bioprinted scaffold with anisotropic structures and heterogeneous components similar to those of the native humeral head. With the dual stimuli of dynamic compression and biochemical cues modulating endochondral ossification, anisotropic regeneration of hyaline cartilage and subchondral bone was achieved. The efficiency of this approach of applying architecture and biochemical and biomechanical cues may have profound implications for large joint tissue engineering in general, resulting in progress in the reconstruction of shoulder joints in animals, as well as other large joints such as the knee, hip, and wrist. This approach also provides an alternative for the design and fabrication of large, anatomically matched implants of clinically relevant sizes. This research furthers our understanding of engineering 3D bioprinted joint scaffolds through the strategic application of biomechanical and biochemical stimuli and anisotropic architecture.

## 5 Experimental Section

### Materials

The hydrogel mixture used as a cell carrier was composed of gelatin (G6411), fibrinogen (F8630), hyaluronic acid, Pluronic F‐127, hydroxyapatite (HA), PCL (molecular weight ≈65 000), and glycerol (G2025); all of which were acquired from Sigma‐Aldrich (St. Louis, MO, USA). PTH was obtained from BD Biosciences (San Jose, CA). Cell culture compositions, such as *α*‐minimal essential medium (*α*‐MEM), phosphate‐buffered saline (PBS), fetal bovine serum (FBS), trypsin, penicillin, and streptomycin, were obtained from HyClone (Carlsbad, CA, USA). Antibodies against COL‐II and COL‐X were obtained from Abcam (Cambridge, UK). The CCK‐8 and live/dead kits were acquired from Beyotime Biotechnology (Shanghai, China). New Zealand rabbits (1‐month‐old, 0.8–1 kg; 3 months old, 3.5–4 kg) were obtained from Jiagan Biological Technology Co., Ltd. (Shanghai China).

### The Design of the Artificial Humeral Head

A 3‐month‐old rabbit was subjected to a 12.7 µm resolution multilayer laser scanner (Berding, Loveland, OH, USA) to obtain images of the humeral head of the forelimb joint (matched with the rabbits used for in vivo arthroplasty). The humeral head was then rebuilt in three dimensions using computer‐aided design. Anatomically matched bioscaffolds were designed according to a previous report.^[^ ^72^ ^]^

### Preparation of Bioinks and Printing Systems

BMSCs were obtained from the tibial marrow of 1‐month‐old rabbits, cultured, and expanded as described previously.^[^ ^73^, ^74^ ^]^ BMSCs at passage three were blended with two different composite solutions at room temperature for the preparation of Bioink‐A (with or without PTH, named Bioink‐A1 or Bioink‐A2, respectively) and Bioink‐B, and then stored at 4 °C. The compositions of the different bioinks used are listed in **Table** **1**. The specific preparation process was performed as described previously.^[^ ^36^ ^]^ The OPUS (Novaprinter, Suzhou, China) with multiple nozzles was used to fabricate the bioscaffold. Specifically, five nozzles loaded with different components were used to prepare 3D humeral architectures. Among these, two low‐temperature controlling nozzles with Bioink‐A (A1: without PTH, A2: with PTH) and Bioink‐B were used for cartilage and subchondral bone printing, respectively. Another low‐temperature control nozzle loaded with a mixture of 33% w/v Pluronic F‐127 in 10% v/v glycerol solution was used as the sacrificial material. Two high‐temperature controlling nozzles with PCL and PCL/HA (80%:20%) were used for the mechanical support of the cartilage and subchondral bone areas, respectively. PCL‐HA (20%) was selected based on previous findings on cell viability, melting point, and osteochondral histogenesis using the same materials.^[^ ^12^ ^]^

**Table 1:** The basic composition of Bio‐ink‐A and Bio‐ink‐B

Composition	Gelatin [mg mL^−1^]	Fibrinogen [mg mL^−1^]	Hyaluronic acid [mg mL^−1^]	Glycerol	PTH	Cell type and density [cells per mL]
Bio‐ink‐A1	45	30	3	10% v/v	/	BMSCs, 40 × 10^6^
Bio‐ink‐A2	45	30	3	10% v/v	10mmol	BMSCs, 40 × 10^6^
Bio‐ink‐B	35	20	3	10% v/v	/	BMSCs, 40 × 10^6^

### 3D Bioprinting of the Artificial Humeral Head

During the printing process, the cell‐laden hydrogels (Bioink‐A1, Bioink‐A2, and Bioink‐B) were printed using low‐temperature nozzles, and the hydrogels were printed through a 300‐µm Teflon nozzle at an air pressure of 60–80 kPa. A 250‐µm metal nozzle was applied to print Pluronic F‐127 under an air pressure of 220–300 kPa. The temperature in the Bioink‐A and Bioink‐B chambers was maintained at 18 °C throughout the printing process. The PCL and PCL/HA polymers were heated at 70 °C for melting and squeezed out through a 250‐µm cone‐shaped metal nozzle under an air pressure of 800 KPa. After printing, the fabricated constructs were crosslinked at 37 °C by adding thrombin solution (20 UI mL^−1^, Sigma‐Aldrich) for half an hour. Then, PBS solution was applied to wash out the Pluronic F‐127 three times. Humeral scaffolds were classified into two different types (with the incorporation of PTH or without the incorporation of PTH).

### Cell Viability Assays

Once fabricated, the printed humeral scaffolds were transferred into a normal culture medium and cultivated in a 5% CO_2_ incubator at 37 °C. The culture medium was changed every 2 days. After 1, 3, 5, and 7 days of cultivation, the cell viability of the printed humeral constructs was assessed using CCK‐8 and a live/dead assay kit in accordance with the manufacturer's instructions. The percentage of live cells and normalized optical intensity were calculated.

### Biomechanical Stimulation

After cultivation in a normalized medium for 24 h, the scaffolds were transferred into a chondrogenic medium and cultured in a custom‐designed bioreactor (Figure S8, Supporting Information). All scaffolds were classified into four different groups: scaffolds without PTH or biomechanical stimulus (named the no stimulus group); scaffolds with PTH but no biomechanical stimulus (named the biochemical stimulus group); scaffolds with biomechanical stimulus but without PTH (named the biomechanical stimulus group); and scaffolds with PTH and biomechanical stimulus (named the double stimuli group). For the scaffolds with biomechanical stimuli, a custom‐built dynamic compression system was applied for periodic dynamic loading to the construct during in vitro culture (Figure S9, Supporting Information) (provided by the Chinese National Tissue Engineering Center). The specific stimulation cycle was 2 MPa for 30 s, 4 MPa for 30 s, and 6 MPa for 30 s. Cycles continued for 60 cycles per day, and the entire stimulation period lasted 8 weeks.

### In Vitro Analysis of Endochondral Ossification

To evaluate the efficiency of different stimuli in the modulation of endochondral ossification in vitro, two cylindrical scaffolds mimicking the structures of cartilage and bone were joined together and cultured in a dynamic compression system, which could simulate the integrated humeral head scaffold, as demonstrated in Figure 3A. Meanwhile, after in vitro stimulation, the top and down cylindrical scaffolds could be extracted and analyzed separately. Thus, the cells from the outer layer and inner layer could be washed out separately to perform RT‐PCR and confocal laser microscopy analysis. After stimulation for 1, 7, and 14 days, the cells on the scaffolds were washed out, and the RNA of the cells from each group was extracted. The expression of the hyaline cartilage‐related marker genes aggrecan (AGG), SOX‐9, and COL‐II; fibrocartilage‐related marker genes COL‐1, COL‐X, and MMP‐13; and osteogenic genes OCN, OPN, and runt‐related transcription factor (RUNX‐2) were evaluated. Primers used for these genes are listed in **Table** **2**. For confocal analysis, after stimulation for 4 weeks, the cells in the scaffolds were stained with antibodies against COL‐II (ab65871, Abcam, Cambridge, MA, USA) and COL‐X (ab49945, Abcam, Cambridge, MA, USA), and nuclei were counterstained with DAPI. Images were captured using a confocal microscope (Leica). The intensities of COL‐II and COL‐X were determined by semiquantitative analysis. After 2 months of stimulation in vitro, the scaffolds from each group were collected, and photos of the scaffolds were taken with a light camera. The scaffolds were then dehydrated in increasing concentrations of alcohol (70–100%) and embedded in polymethyl methacrylate. Coronal sections of the central region of each scaffold (≈100 µm thick) were cut and polished to a final thickness of ≈20–30 µm. Samples from each group were stained with HE, Masson, SO/FG, TB, and AB staining to identify new tissue formation.

**Table 2:** Primer sequence of genes for RT‐PCR

Gene	Forward primers (from 5ʹ to 3ʹ)	Reverse primers (from 5ʹ to 3ʹ)
*Gapdh*	AGGTCATCCACGACCACTTC	GTGAGTTTCCCGTTCAGCTC
*Aggrecan*	ACCGAGGTCAGTGGATTGTC	CCAGGTCAGGGATTCTGTGT
*SOX‐9*	GGTGCTCAAGGGCTACGACT	GGGTGGTCTTTCTTGTGCTG
*COL‐II*	*CAACAACCAGATCGAGAGCA*	*GCTCCACCAGTTCTTCTTGG*
*COL‐1*	*ACATCCCGCCAGTCAC*	*CACGTCATCGCACAAGA*
*COL‐X*	*GAAAACCAGGCTATGGAACC*	*GCTCCTGTAAGTCCCTGTTGTC*
*MMP‐13*	*TCAAGATGCAGCCAGGTGTC*	*AACTAAGCTTTGCCCTGAA*
*OCN*	*GGCCAGGCAGAGGCAAAG*	CTCCAGGGGATCCGGGTAA
*OPN*	TCTCAGAAGCAGAATCTCCTAAC	ATGGCTTTCAATGGACTTACTC
*RUNX‐2*	*TTTAGGGCGCATTCCTCATC*	*GGACTTGGTGCAGAGTTCA*

### Surgical Joint Replacement

The animal experimental protocols were in accordance with the Guide for the Care and Use of Laboratory Animals published by the National Academy Press and approved by the Animal Care and Experiment Committee of the Ninth People's Hospital, Shanghai Jiao Tong University School of Medicine. In total, 40 New Zealand white rabbits were randomly divided into four groups (*n* = 10 per group): no stimulus group, biochemical stimulus group, biomechanical stimulus group, and double stimuli group. Ten rabbits served as the normal control (NC) group. After anesthesia, a craniolateral approach for complete exposure of the humeral head was established, as reported previously.^[^ ^72^ ^]^ An osteotomy along the metaphysis junction was performed to remove the humeral head while preserving the greater and lesser tubercles and all attached soft tissue attachments to simulate semi‐shoulder arthroplasty (Video S4, Supporting information). Thereafter, an anatomically matched bioscaffold was implanted by press fitting. The osteotomy and arthroplasty processes are shown in Figure S10, Supporting Information. The joint capsule was then sutured and the infraspinatus and deltoid tendons were reattached. The subcutis was stitched with 4‐0 polydioxanone sutures followed by skin closure. Antibiotics were administered intramuscularly for prophylactic infection. After surgery, the rabbits were allowed to move freely in their cages and were fed standard food and water. For the inflammatory response, serum from each group was collected at different time points (1, 2, 4, 8, and 12 weeks) and quantified by IL‐1*β* and TNF‐*α* concentrations (*n* = 6 for each group), using enzyme‐linked immunosorbent assay (ELISA) kits according to the manufacturer's instructions. Locomotion and weight bearing were assessed 4 weeks post‐surgery.

### X‐ray and MRI Analysis

To detect the fixation and regeneration of humeral head restoration, X‐rays and MRI (7.0 T system, Bruker PharmaScan; Bruker BioSpin, Germany) were performed 16 weeks after the operation. The experimental procedure was as follows: the experimental animals were anesthetized with pentobarbital (concentration: 2%, 2 mL kg^−1^) and then placed on an X‐ray camera for the front and side photographs, after which the experimental animals were subjected to MRI. The T1, T2, and proton density (PD) mapping sequences were acquired using the following imaging parameters as previously described: ^[^ ^75^ ^]^ repetition time (TR), 1000 ms; echo time (TE) 13.8 ms, bandwidth 227 (BW, Hz/pixel); flip angle, 180°; field of view, 80 mm; matrix, 512 × 256; and slice thickness, 2 mm with a 20% distance factor. After the experiment was completed, the data were exported and analyzed to assess the fixation and regeneration of the bioprinted scaffolds.

### Histology and Histomorphometric Analysis of In Vitro and In Vivo Samples

4 months after in vivo implantation, the experimental animals were euthanized, and humeral head samples from each group were collected. A gross view of the humeral head was captured using a high‐resolution camera. After sagittal dissection, the cartilage thickness was calculated as the linear distance from the articular surface to the subchondral bone at ten equidistant locations throughout the total articular cartilage. Thereafter, some samples were processed for hard tissue slicing, as described in Section 2.7, and SO/FG staining was performed to identify the developmental process of endochondral ossification and new cartilage formation. HE, Masson and VG staining were performed to identify the regeneration of new bone in the inner area of the scaffolds. The remaining specimens were decalcified in 0.5 m EDTA solution, embedded in paraffin, and processed according to standard histological protocols. Immunohistochemical staining with Col‐II (green) and Col‐X (red) was performed to further evaluate the expression of endochondral ossification‐specific proteins in the newly formed cartilage tissue. In addition, the ratio of cartilage to the total cartilage area was measured. The BV/TV was measured in the sliced bone tissue.

### Biomechanical Analysis

The remaining retracted specimen was pruned to a cylindrical shape with a diameter of 5 mm. Thereafter, a testing system (Instron, Grove City, PA, USA) was applied to evaluate the maximum failure load, according to previous report.^[^ ^76^ ^]^

### Statistical Analysis

All statistical analyses were conducted using SPSS software. Statistical significance was set at *p* < 0.05. One‐way analysis of variance was used for statistical analysis, followed by the least significant difference post‐hoc test to compare selected data pairs.

## Conflict of Interest

The authors declare no conflict of interest.

## Author Contributions

T.L., Z.M., Y.Z., and Z.Y. contributed equally to this work. Conceptualization: T.L., K.D., X.C., J.W., and J.Z. Methodology: T.L., Z.Y., X.Z., J.Z, and K.D. Investigation: T.L., Z.M., H.H., Z.Y., Y.R., Y.Z., Y.L., W.W., D.L., W.L., L.Q., T.W., and Y.M. Visualization: Z.M. and Z.Y. Supervision: K.D., X.C., J.W., and J.Z. Writing—original draft: T.L., Z.M., H.H., Y.Z., and X.Z. Writing—review and editing: T.L., Z.M., H.H., Z.Y., K.D., J.W., and X.C.

## Supporting information

Supporting Information

Supplemental Video 1

Supplemental Video 2

Supplemental Video 3

Supplemental Video 4

## Data Availability

The data that support the findings of this study are available on request from the corresponding author. The data are not publicly available due to privacy or ethical restrictions.
