# Supplementary material for: Regeneration of Humeral Head Using a 3D Bioprinted Anisotropic Scaffold with Dual Modulation of Endochondral Ossification
Source: Adv Sci (Weinh). 2023 Feb 8;10(12):2205059. doi: 10.1002/advs.202205059 (PMC10131811; doi:10.1002/advs.202205059)
Supplement: Supplementary file 1 — Supporting Information [file ADVS-10-2205059-s004.pdf]

ADVANCED SCIENCE
Open Access

Supporting Information

for *Adv. Sci.*, DOI 10.1002/advs.202205059

Regeneration of Humeral Head Using a 3D Bioprinted Anisotropic Scaffold with Dual Modulation of Endochondral Ossification

Tao Li, Zhengjiang Ma, Yuxin Zhang, Zezheng Yang, Wentao Li, Dezhi Lu, Yihao Liu, Lei Qiang, Tianchang Wang, Ya Ren, Wenhao Wang, Hongtao He, Xiaojun Zhou, Yuanqing Mao, Junfeng Zhu*, Jinwu Wang*, Xiaodong Chen* and Kerong Dai**

Regeneration of humeral head using a 3D bioprinted anisotropic scaffold with dual modulation of endochondral ossification

Authors

Tao Li[1, 2, #], Zhenjiang Ma[1, #], Yuxin Zhang[3, #], Zezheng Yang[4, #], Wentao Li[1], Dezhi Lu[5], Yihao Liu[1], Lei Qiang[6], Tianchang Wang[1], Ya Ren[6], Wenhao Wang[6], Hongtao He[7], Yuanqing Mao[1], Xiaojun Zhou[8, *], Junfeng Zhu[2, *], Jinwu Wang[1,*], Xiaodong Chen[2, *], Kerong Dai[1, *]

1 Shanghai Key Laboratory of Orthopaedic Implant, Department of Orthopaedic Surgery, Shanghai Ninth People's Hospital Affiliated Shanghai Jiao Tong University School of Medicine, 639 Zhizaoju Rd, Shanghai 200011, China.

2 Department of Orthopaedics, Xinhua Hospital affiliated to Shanghai Jiaotong University School of Medicine, No. 1665 Kongjiang Road, Shanghai, 200092, P. R. China.

3 Department of Oral Surgery, Shanghai Ninth People's Hospital, Shanghai Jiao Tong University School of Medicine; College of Stomatology, Shanghai Jiao Tong University; National Center for Stomatology; National Clinical Research Center for Oral Diseases; Shanghai Key Laboratory of Stomatology, Shanghai 200011, China.

4 Department of Orthopedics, The Fifth People's Hospital of Shanghai, Fudan University, Minhang District, Shanghai 200240, P. R. China.

5 School of Medicine, Shanghai University, Jing An District, Shanghai 200444, China.

6 Southwest JiaoTong University College of Medicine, No. 111 North 1st Section of Second Ring Road, Chengdu, 610036, China.

7 The Third Ward of Department of Orthopedics, The Second Hospital of Dalian Medical University, No. 467, Zhongshan Road, Shahekou District, Dalian, Liaoning Province 116000, P. R. China.

8 College of Biological Science and Medical Engineering; State Key Laboratory for Modification of Chemical Fibers and Polymer Materials, Donghua University, Shanghai 201620, P. R. China.

[#] Tao Li, Zhenjiang Ma, Yuxin Zhang and Zezheng Yang equally contributed to this work.

[*] Kerong Dai, Xiaodong Chen, Jinwu Wang, Junfeng Zhu and Xiaojun Zhou are corresponding authors.

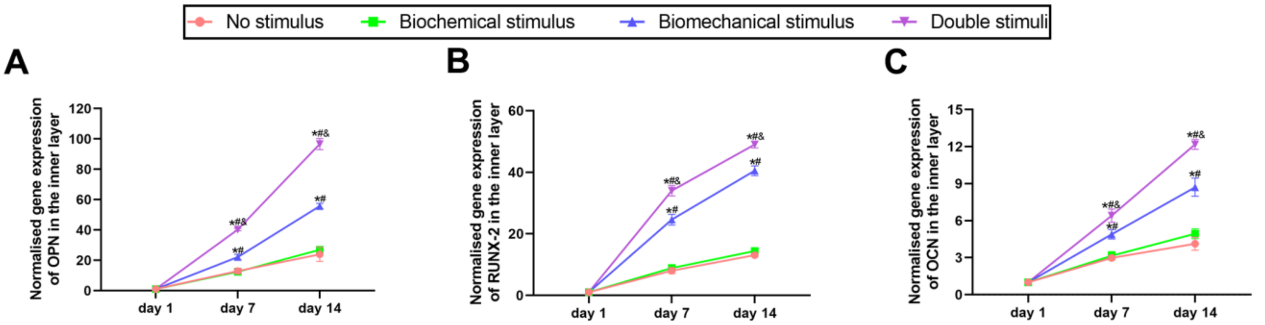

Figure S1. The expression level of osteogenesis-related genes. (A) OPN, **(B)** RUNX-2, and **(C)** OCN in the subchondral bone area in each stimulation group. An * indicates a P-value < 0.05 compared with the native humeral group. A # indicates a P-value < 0.05 compared with the biochemical stimulus group. An & indicates a P-value < 0.05 compared with the biomechanical stimulus group.

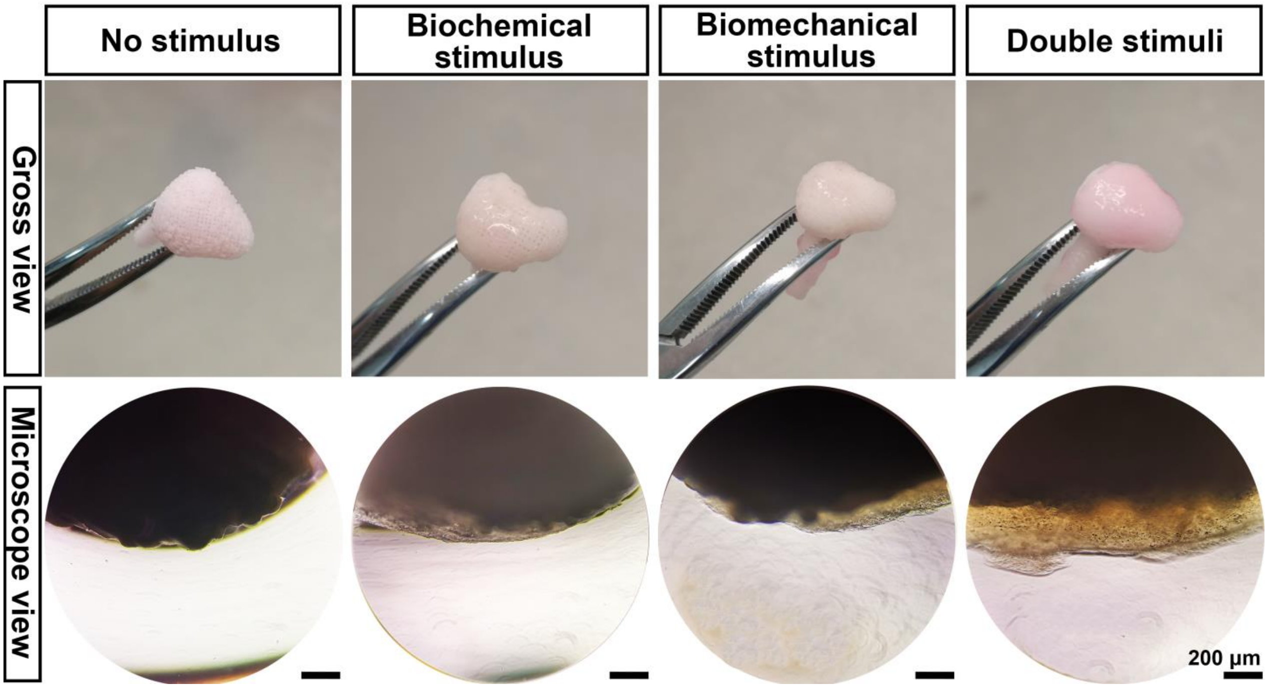

Figure S2. Gross observation and microscopic observation of stent surface morphology after humeral head stent stimulation in each group.

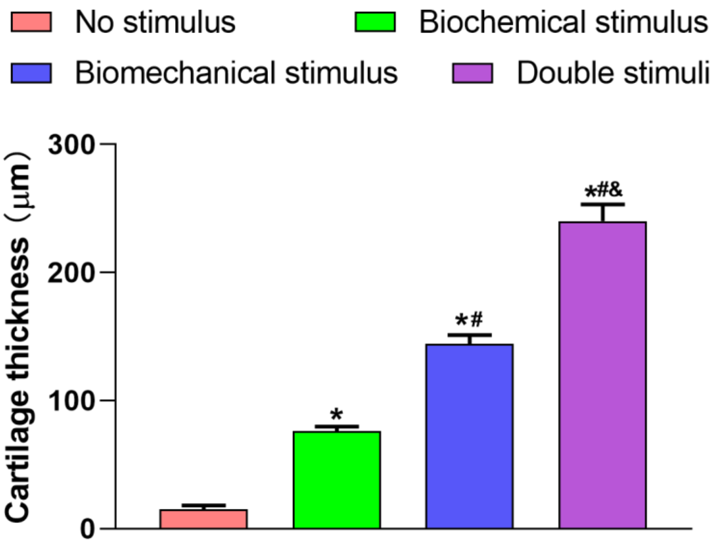

Figure S3. Quantification analysis of cartilage thickness in the outer layer after in vitro stimulation for 2 months. An * indicates a P-value < 0.05 compared with the native humeral group. A # indicates a P-value < 0.05 compared with the biochemical stimulus group. An & indicates a P-value < 0.05 compared with the biomechanical stimulus group.

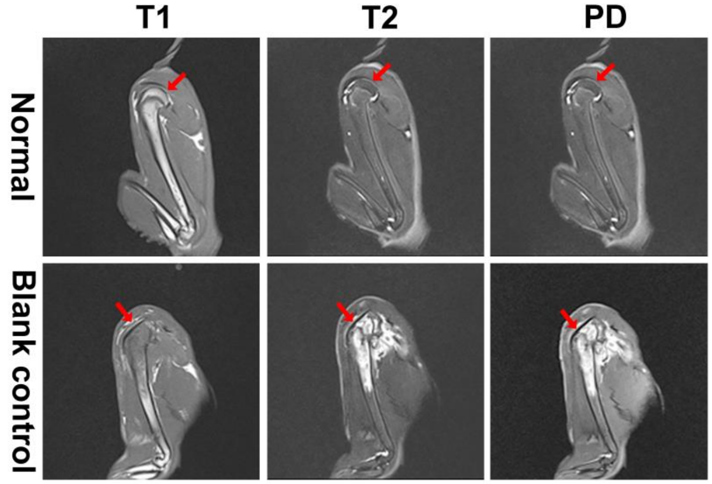

Figure S4. MRI photos of the shoulder joint in the normal group and blank control group. (T1: T1 weighted imaging, T2: T1 weighted imaging, PD: proton density weighted image).

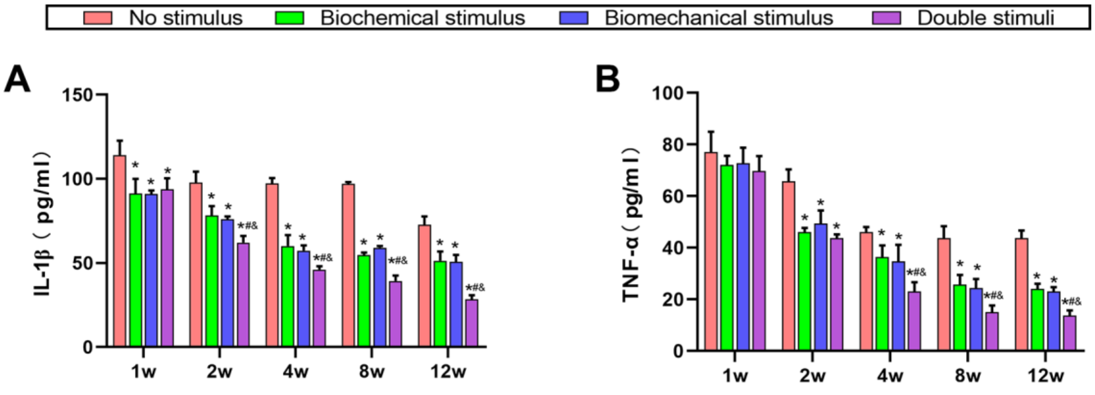

Figure S5. Quantification analysis of IL-1β and TNF-α in the serum of surgical rabbits at 1, 2, 4, 8, and 12 weeks after surgery. (A) Quantification analysis of IL-1β. **(B)** Quantification analysis of TNF-α. An * indicates a P-value < 0.05 compared with the no stimulus group. A # indicates a P-value < 0.05 compared with the biochemical stimulus group. An & indicates a P-value < 0.05 compared with the biomechanical stimulus group.

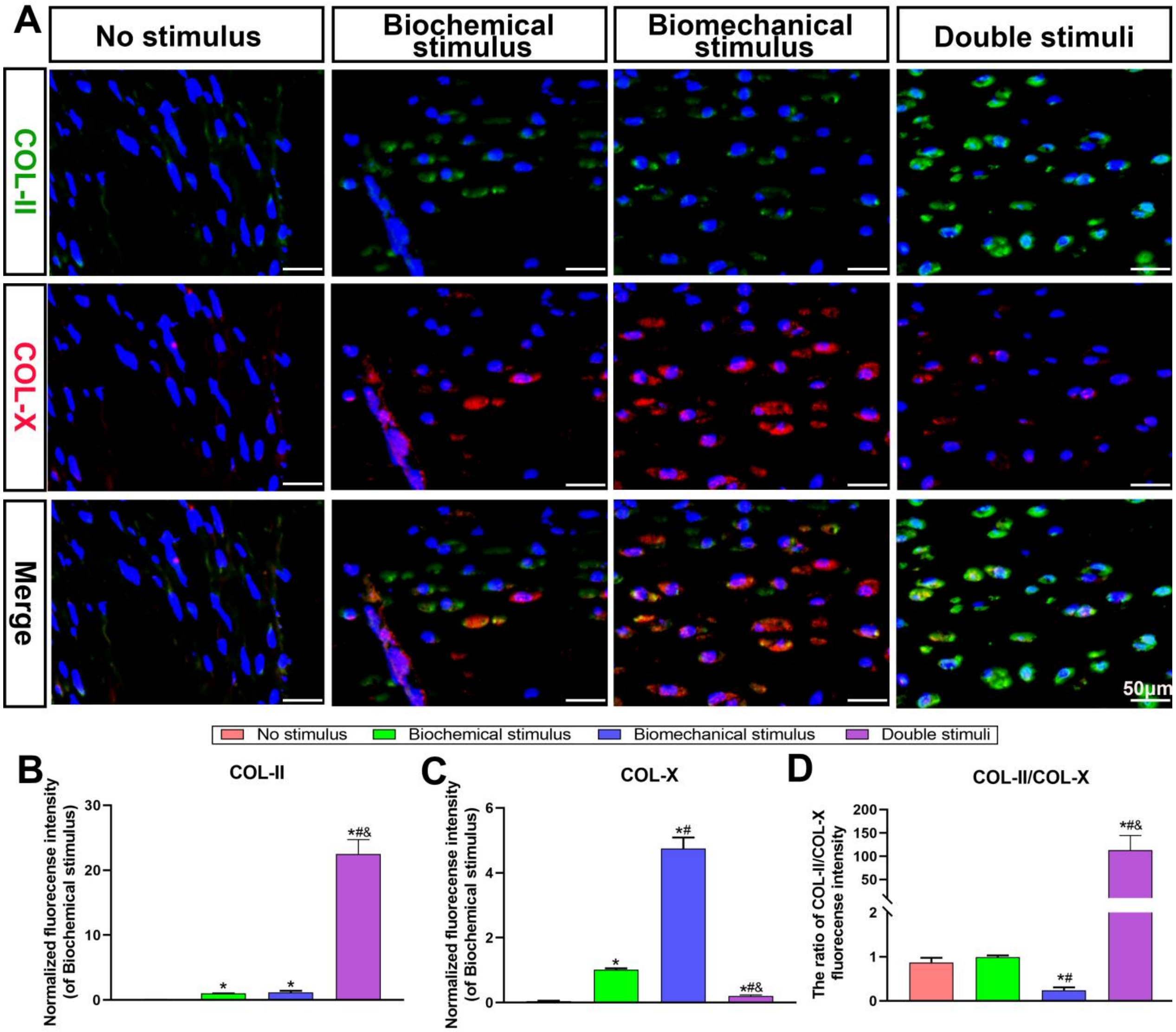

Figure S6. Analysis of COL-II and COL-X in the outer layer of the regenerated humeral head. (A) The expression of COL-II and COL-X as detected by confocal microscopy. **(B)** The quantification of COL-II, **(C)** COL-X, and **(D)** The ratio of COL-II/COL-X. Green: COL-II; red: COL-X; blue: DAPI. An * indicates a P-value < 0.05 compared with the native humeral group. A # indicates a P-value < 0.05 compared with the biochemical stimulus group. An & indicates a P-value < 0.05 compared with the biomechanical stimulus group.

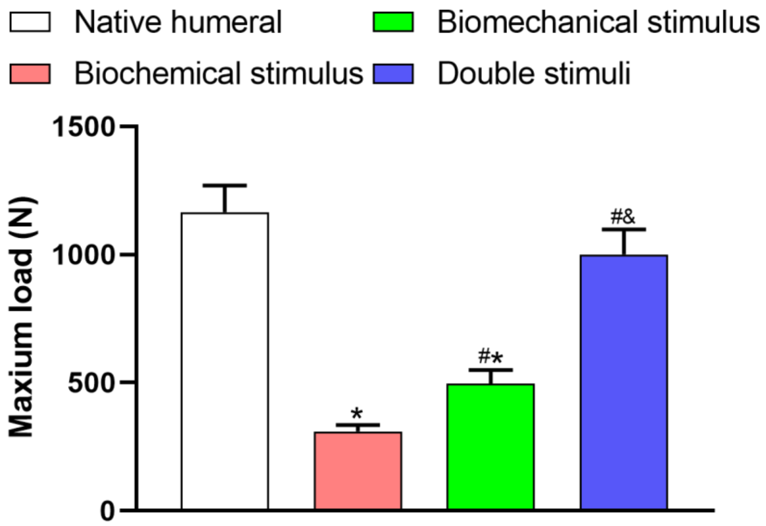

Figure S7. The maximum load of the native humeral head, head from the biochemical stimulus group, head from the biomechanical stimulus group, and head from the double stimuli group. An * indicates a P-value < 0.05 compared with the native humeral group. A # indicates a P-value < 0.05 compared with the biomechanical stimulus group. An & indicates a P-value < 0.05 compared with the biochemical stimulus group.

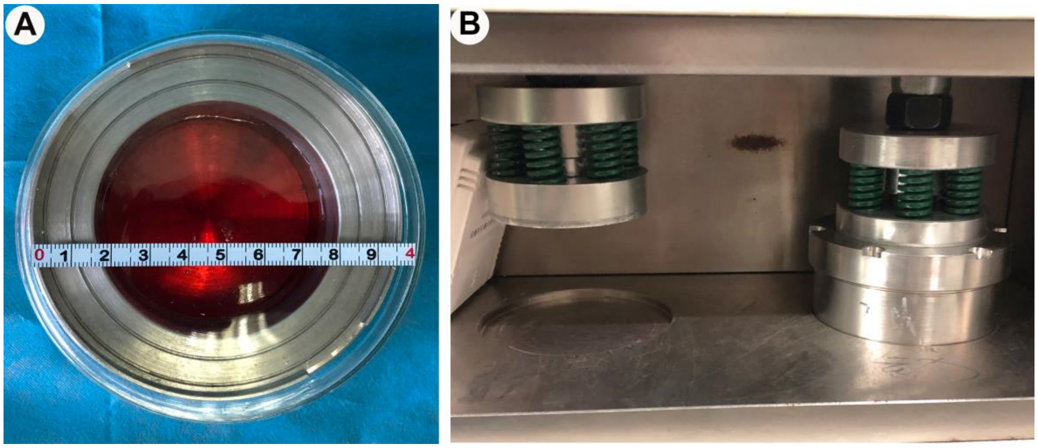

Figure S8. The gross view of the customized bioreactor. **(A)** the customized bioreactor. **(B)** the bioreactors are under dynamic compression.

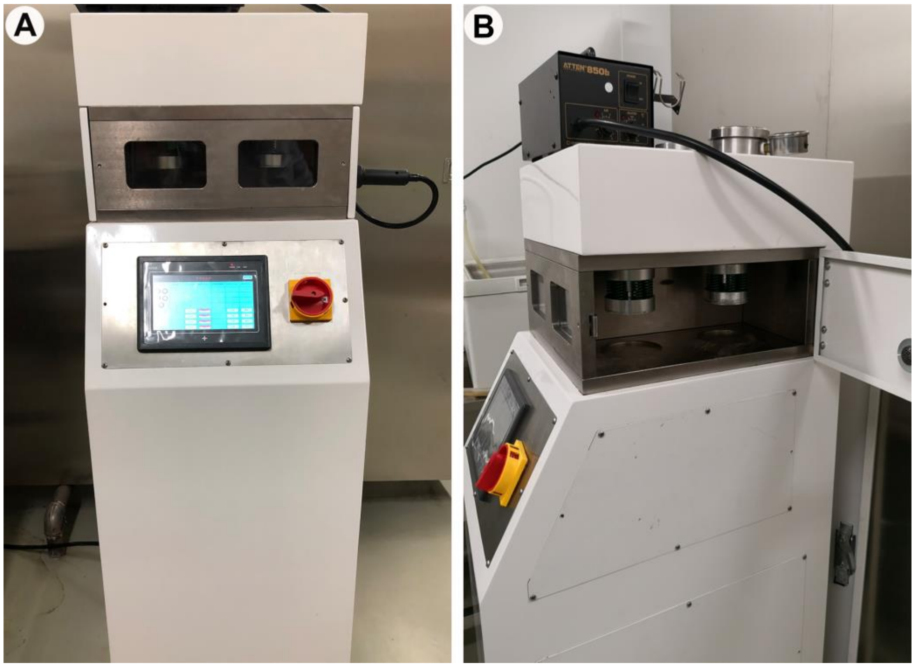

Figure S9. The gross view of the dynamic compression control system. (A) the anterior view. **(B)** the lateral view.

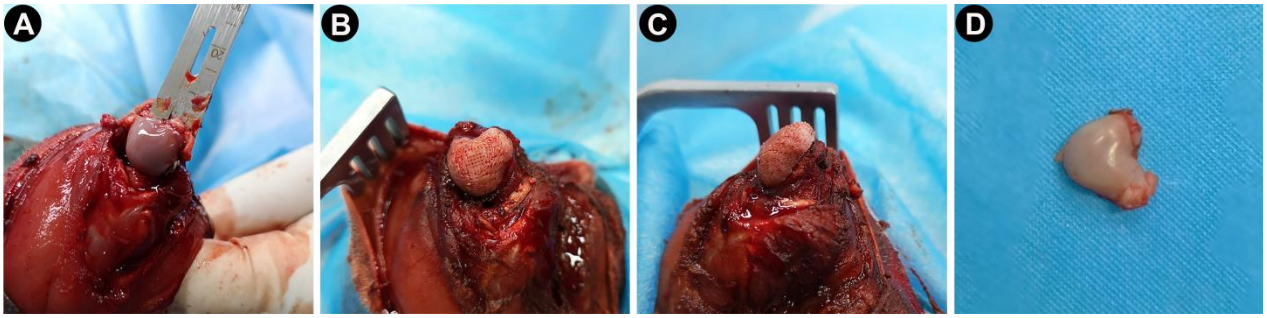

Figure S10. The gross view of humeral head arthroplasty in rabbits with fabricated scaffolds. (A) Osteotomy of the humeral head. **(B)** A front view of the implanted scaffold after arthroplasty. **(C)** Lateral view of the implanted scaffold after arthroplasty. **(D)** The view of the native humeral head after arthroplasty.

Video 1. The fabrication process of a 3D bioprinted hemispherical scaffold with the OPUS system.

Video 2. The movements of rabbits from the double-stimuli group at one month post-operation.

Video 3. The movements of rabbits from the double-stimuli group at four months post-operation.

Video 4. The osteotomy of the humeral head of the rabbit.
